# Comprehensive Studies of Different Cancer Diseases among Less-Developed Countries

**DOI:** 10.3390/healthcare10030424

**Published:** 2022-02-24

**Authors:** Mohammed M. Rahman, Firoz A. D. M. Opo, Abdullah M. Asiri

**Affiliations:** 1 Department of Chemistry, Faculty of Science, King Abdulaziz University, P.O. Box 80203, Jeddah 21589, Saudi Arabia; amasirikau@gmail.com; 2 Center of Excellence for Advanced Materials Research (CEAMR), King Abdulaziz University, P.O. Box 80203, Jeddah 21589, Saudi Arabia; 3 Department of Biomedical Science, College of Natural Sciences, Chosun University, Gwangju 309, Korea; fadmopo@gmail.com; 4 Department of Pharmacy, University of Asia Pacific, 74/A, Green Road, Farmgate, Dhaka 1215, Bangladesh

**Keywords:** carcinoma, cancer, formalin, smoking, food habit, diseases

## Abstract

Recently, the rate of cancer deaths in less-developed countries such as Bangladesh has significantly increased day by day, making it a major health issue. The most predominant types of cancers among the populations of less-developed countries (especially Bangladesh) are lung, throat, colon, gastric, ovarian, breast, and skin cancers. The mortality rate is increasing for both males and females. The main common factors are smoking, use of tobacco leaves, bacterial or viral infection, hereditary disorders, food adulterations, and environmental factors, which are highly responsible for the development of carcinoma in the young to adult population in this region. Raising consciousness among people regarding early diagnosis, decreasing the use of chemicals such as formalin for food preservation, and reducing environmental pollution such as arsenic as well as air pollution might help to reduce the number of deaths. Education and public campaigns can also reduce the intensity of cancer occurrence. Breast, esophagus, and cervical cancer are common diseases in less-developed countries such as Bangladesh.

## 1. Introduction

Globally, cancer is responsible for killing more people than HIV, malaria, and TB combined [1]. Lower-income and lower-middle-income countries are mainly affected by these deadly diseases. In 1960, the rate was almost 25%, and in 2010 it reached nearly 55% in these countries [2,3]. It is assumed that by 2030, more than 13 million people will die of cancer every year, 9 million of them in developing countries [4]. The most predominant diseases are prostate, colorectal, lung, breast, stomach, and liver cancers [5]. Statistically, the ratios differ between males and females; in Bangladesh, lung carcinomas are prevalent in males and breast and cervical cancer are prevalent in females [6]. Each year many people die due to prostate, liver, breast, lung, gastric, and hepatocellular carcinoma, which predominate over other cancers [7]. In Bangladesh, lung, cervical, and breast cancers account for almost 38% of all cancers [8]. Since most cases of cervical carcinoma in Bangladesh are diagnosed and treated at late stages [9], the survival rate is low [8]. In 2010, 55% of people were affected and by 2030, 9 million may die from this disease [9].

**Figure 1:**
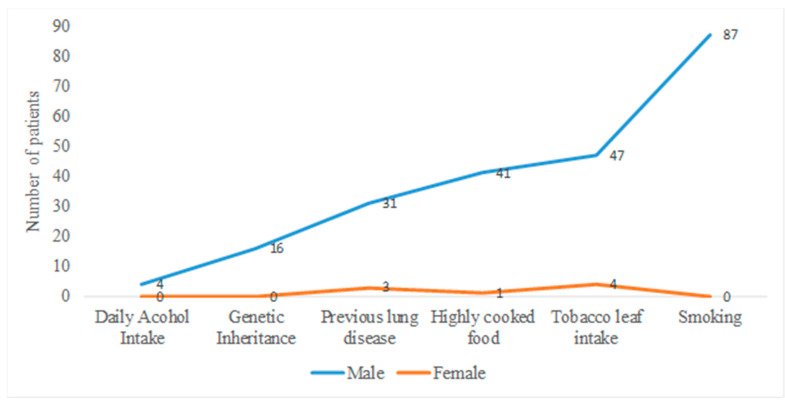
Line graph showing prevalence of lung cancer among Bangladeshi males and females. For males, smoking is the most common contributing factor in cancer development, and for females it is tobacco leaf intake. Men are more susceptible to lung cancer than women, as the rate of smoking is higher rate among men, but tobacco leaf use is increasing lung carcinoma rates among women.

The number of cancer patients is drastically increasing day by day. Figure 1 shows the scenario of patients affected by and dying from cancer diseases. In 2008, there were 1.61 million deaths from lung cancer, 1.38 million from breast cancer, and 1.38 million from colon cancer. Smoking, hepatitis B and C virus, *Helicobacter pylori* bacterial infection, water contamination by arsenic, the use of carcinogenic chemicals, and additives in food items are the major elements causing cancer in less-developed countries such as Bangladesh [10]. The rising number of prostate cancer cases among the Bangladeshi population is due to gene polymorphism, age, and genetic heredity, even though the genetic relation is controversial [11,12]. In 2018, more than 750 people died due to prostate cancer in Bangladesh [13]. This investigation focused on the causes mentioned above for the rising cancer rate among Bangladeshi people.

## 2. Methods of Data Collection and Assessment

In our review, we collected data based on the PRISMA systemic review guidelines (Supplementary Table S1) [14]. We found that the number of cancer patients globally increased significantly each year, including in less-developed countries such as Bangladesh. We introduced a dataset for obtaining policies regarding cancer patients in this investigation. We searched the data based on specific cancers in published papers and renowned newspapers in Bangladesh. After that, we screened and focused on published papers from the beginning of 2000 to 2021. Later, the collected papers were again scrutinized with the help of online software to determine whether they were real or fake journals (Supplementary Table S2). Predatory journals were canceled at this stage, and we sought to obtain information from PubMed, an internationally recognized database. We also carried out a search for papers by using Google Scholar, focused on several parameters related to causes (age, sex, food pattern, economic condition, area of residence). Prevalence data were obtained by searching the number of patients affected in a specific period, and statistical analysis was performed by using ANOVA and Microsoft Excel databases. The values were taken from different papers, and the mean value was calculated for the statistical data. For the final assessment, we cross-checked each author/paper from the source and collected the data based on parameters such as patient numbers and causes of the cancers.

## 3. Results

### 3.1. Cause of Lung Cancer

To maintain freshness, the chemical formalin is extensively used for fruits, fish, and vegetables. Formalin-treated foods are openly sold in markets in less-developed countries, especially Bangladesh. Ingesting these foods may cause different types of cancer, predominantly lung cancer [15]. Smoking, tobacco use, previous history of lung disease such as asthma or tuberculosis, and genetic factors are the key factors in the development of lung cancer [16]. Polymorphic changes in the MDM2 gene have been identified as another reason for lung cancer, based on data collected from more than 11,638 patients [17]. Age is another important parameter. People older than 65 years of age are significantly affected compared to younger people in less-developed countries. It was reported that about 5887 lung cancer patients were admitted to the hospital in 2020, especially the National Institute of Cancer Research and Hospital (NICRH). Experts have suggested that smoking and air pollution are the main reasons for lung carcinoma [18]. Family income, area of residence, education status, and marital status are the four most common contributing factors in lung cancer, mainly for men older than 55 [19].

#### Prevalence of Lung Cancer

In 2012, an estimated 14.1 million people were affected by cancer and about 8.2 million died [7]. Among them, 17.8% died due to lung cancer. This rate was comparatively higher than stomach and liver cancers [20,21]. A study was conducted of 104 male and female individuals (about 94.20 and 5.80%, respectively) in different areas of Bangladesh. Important risk factors for developing lung cancer in Bangladesh were identified. Table 1 shows the risk factors from lower to higher, based on the data (Figure 1). The following order was shown for males—smoking > previous history of lung disease > highly cooked food > genetic inheritance > tobacco leaf intake > alcohol consumption; while the following order was shown for females—genetic inheritance < highly cooked food < previous history of lung disease < tobacco leaf intake [22]. Every year, more than 190,000 people are affected by lung cancer in Bangladesh. Approximately 30,000 patients are expected to die from this cancer [23,24]. A report published in 2020 indicated that among 400 cancer patients in 2016, 11 patients were affected by lung cancer, with a male-to-female ratio of 10:1. According to the report, the highest-selling lung cancer drugs, including cisplatin, gefitinib, and osimertinib, are being produced by top-ranked pharmaceutical companies in Bangladesh [24].

### 3.2. Cause of Liver Cancer

Hepatocellular carcinoma (HCC), i.e., liver cancer, is the third most common cancer, behind lung and gastric carcinoma [25]. About 8 million people in Bangladesh are infected with chronic hepatitis B or C virus. This can proceed to HCC, and recently non-alcoholic fatty liver disease (NAFLD) has been shown to increase the trend toward liver cancer [26,27]. For the manufacturing and processing of sutki (the local name for dried fish), dichlorodiphenyltrichloroethane (DDT) is commonly used, although it is banned by the government. This is also responsible for liver cancer [15].

#### Prevalence of Liver Cancer

**Figure 2:**
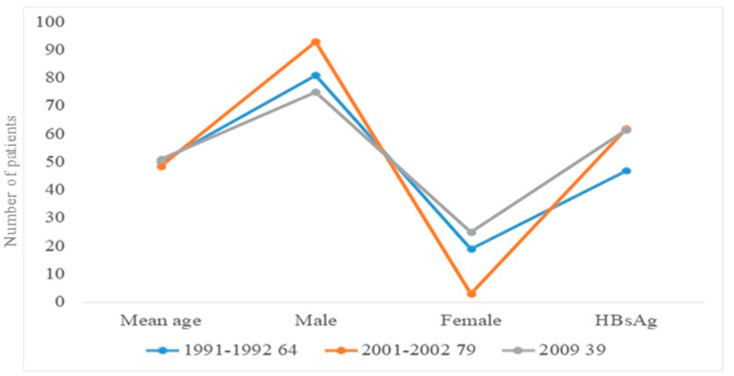
Scenario of hepatocellular carcinoma in Bangladeshi males and females based on age, sex, and HBsAg. Liver carcinoma from 2001 to 2002 was more prevalent in males than in females. A study of 39 male and female patients found the same incidence of HBsAg-related liver carcinoma in males and females.

Hepatocellular carcinoma is usually a male-predominant disease in Bangladesh, affecting men between the ages of 41–92 years [28]. A study was conducted by Karim et al. at Dhaka Medical College of 79 patients with hepatocellular carcinoma, based on age, sex, and HbsAg (Figure 2). Another study found a male predominance for this disease and concluded that HBV was responsible for 61.5% of cases of HCC in Bangladesh [28,29]. The average age of hepatocellular carcinoma patients in Bangladesh is 41–92 years [30]. According to a WHO report in 2018, the number of hepatocellular carcinoma cases was 3112 and the mortality rate was 2.68% [31]. Current treatment includes the use of sorafenib and pegylated interferon alpha, as most liver cancer is related to hepatitis infection. The hepatitis B core antigen-based vaccine is gaining popularity for prevention of liver carcinoma [6].

### 3.3. Cause of Breast Cancer

Female gender, age, obesity, menarche (under 12 years of age), and radiation therapy to the chest or breasts are the main factors for breast cancer (BC) development [32,33,34]. One prevalent major risk factor among women was found to be overweight. In addition, menarche, contact with radiation to the chest or face, and age under 40 years were identified as major risk factors among Bangladeshi women (Table 2). Family history is also a predominant factor in breast cancer [35]. The tendency to avoid breastfeeding and the changing reproductive system may increase the risk of breast cancer [36]. With the lack of accessibility to a hospital and the cost of diagnosis, many women are unwilling to seek a diagnosis due to their socio-economic status. Improper treatment, poverty, and late diagnosis are also contributing factors [37,38]. A report published by NICRH indicated that illiterate housewives were highly prone to be affected by breast cancer. Women living in urban areas are different from women in rural areas in their reproductive behavior; they are reluctant to marry, have children, or breastfeed [39]. The use of chemicals in dermatological products and crop production and bisphenol A in plastic materials is changing the secretion of estrogen, also leading to breast cancer development [38].

#### Prevalence of Breast Cancer

The incidence of breast cancer in developing countries is increasing significantly day by day. It has been estimated that more than 1.67 million people were identified as breast cancer patients [40], especially premenopausal women [41]. One of the biggest cancer-based hospitals in Bangladesh is the National Institute of Cancer Research and Hospital (NICRH). The hospital conducted a study from 2005–2010 among 5255 breast cancer patients in different age groups, from 15 to 94 years (Table 3). More than 56% of women were affected at reproductive age, between 15 and 44 years [10,42]. The incidence is higher due to a lack of awareness, and in most cases, women are affected at a young age [43]. The most common anti-cancer drugs prescribed for breast cancer are carboplatin, 5-fluorouracil, docetaxel, and doxorubicin, according to data obtained from different pharmacies in Dhaka city [24].

### 3.4. Cause Cervical Cancer

In less-developed countries, the most common gynecological cancer is cervical cancer [44], which is one of the leading causes of cancer death of women in Bangladesh. More than 50 million women are at risk for cervical cancer, with 17,686 cases diagnosed and 10,362 deaths each year [45]. Factors related to sex and reproduction are directly associated with cervical cancer, such as young age at the time of first sexual intercourse, multiple sexual partners, and unhygienic sex. Human papillomavirus (HPV) was also reported to be responsible for cervical cancer. The prevalence of cervical cancer in Bangladesh has been reported to be 25–30 per 100,000 women [46].

#### Prevalence of Cervical Cancer

**Figure 3:**
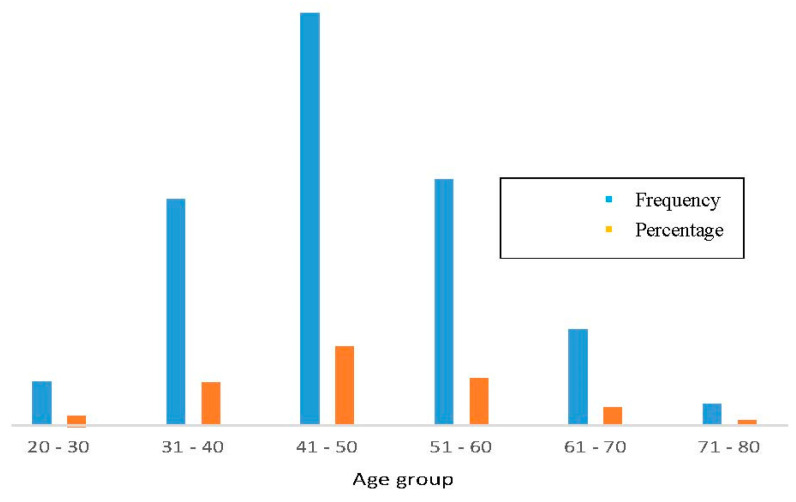
Incidence of cervical cancer in different age groups. Highest rate in 41-to-50-year-old group, and lowest in 71-to-80-year-old group. Green indicates frequency and brown indicates percentage. Most susceptible age group is 41–50 years; females in 31-to-40-year-old and 51-to-60-year-old groups had same ratio of cancer in Bangladesh.

**Figure 4:**
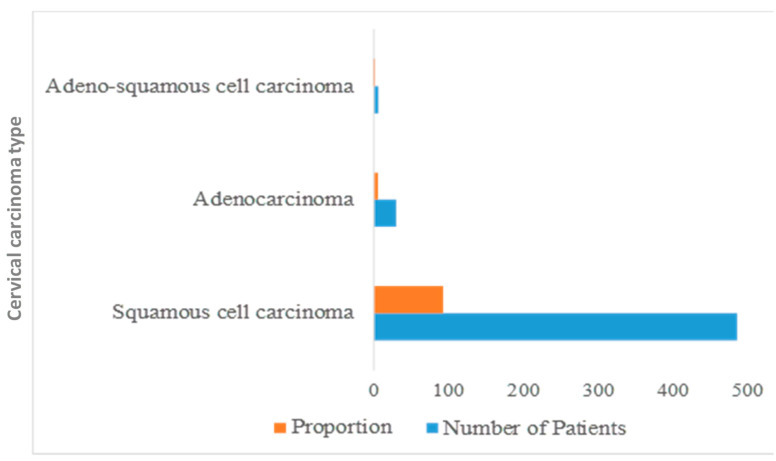
Types of cervical carcinoma. Adenosquamous cell carcinoma is present in a small proportion, while squamous cell carcinoma is more predominant in Bangladeshi people.

A study was conducted at the Delta Medical College and Hospital in Bangladesh among 2264 female cancer patients. A total of 23% of patients (523 out of 2264) were diagnosed with cervical carcinoma. Based on incidence, the majority (39.38%) of cervical cancer patients were in the 41-to-50-year-old age group (Figure 3). In most cases, the diagnosis was squamous cell carcinoma, followed by adenocarcinoma and adenosquamous cell carcinoma; the squamous cell carcinoma was the more predominant type (Figure 4). According to the report, nearly 500 people were affected by squamous cell carcinoma. Figure 4 shows the different types of cervical carcinoma with their percentages [47]. For the treatment of cervical carcinoma, surgery, radiotherapy, ifosfamide, paclitaxel, and cisplatin are commonly using in Bangladesh [48].

### 3.5. Cause of Gastric Cancer

In less-developed countries, *Helicobacter pylori* (*H. pylori*) infection is high due to poor socioeconomic conditions [49] compared to Europe and the United States [50]. *H. pylori* causes noncardiac gastric carcinoma and low-grade B-cell mucosa-associated lymphoid tissue lymphoma (MALT) [51], but the vast majority is noncardiac gastric carcinoma [52]. *H. pylori* infection affects people based on age and sex, as well as salt intake, smoking, education, family income, and drinking water [53]. *H. pylori* is an important contributing factor in increasing gastric carcinoma and other gastric malignancies in Bangladesh [54]. 

#### Prevalence of Gastric Cancer

**Figure 5:**
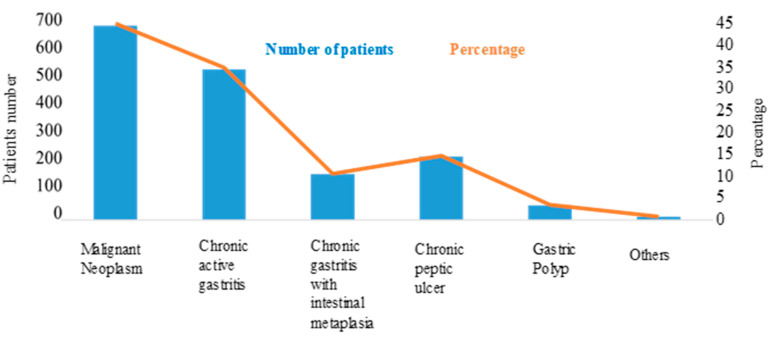
Incidence of different types of gastric carcinoma among Bangladeshi population. Chronic active gastritis was a major cause of gastric cancer, after malignant neoplasm. Untreated gastritis can lead to ulcers; thus, chronic peptic ulcers are in third position for gastric carcinoma.

The *H. pylori* infection rate in Bangladesh is comparatively high (92%) compared to India [55], Thailand [56], and Vietnam [57], which have been reported to be especially high at 81, 74, and 75%, respectively. From January to December 2007, a study was carried out with 1546 patients, and among them, different carcinomas were detected in 636 patients, with a prevalence of gastric adenocarcinoma in 625 of the 636 patients (Figure 5) [54].

### 3.6. Cause of Prostate Cancer

According to the report, age as the main cause of prostate cancer applies to the 46-to-70-year-old group [11]. The rate of prostate cancer is higher for smokers than non-smokers in less-developed countries, especially in Bangladesh. While it has been difficult to identify the genetic causes of prostate cancer due to the lack of an accurate database, it is also difficult to obtain family histories due to illiteracy and the lack of proper screening in rural areas. However, it has been identified that family history plays a role in this cancer [11,58]. Polymorphic changes among two genes CDH1 (-160C/A) and Exo1 (K589E), have been identified as a crucial parameter for the development of prostate carcinoma, as reported in a study of 100 patients [12]. 

#### Prevalence of Prostate Cancer

**Figure 6:**
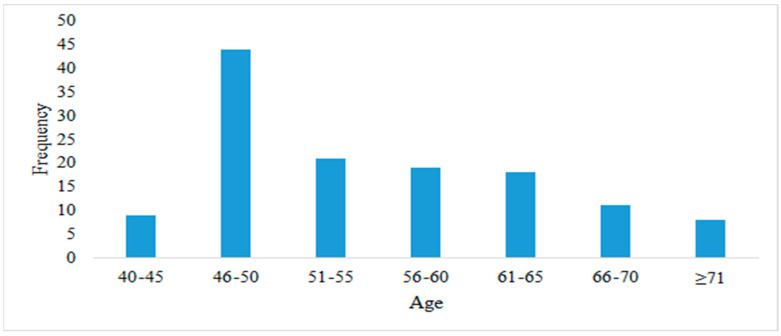
Number of patients by age. Susceptibility to prostate cancer is high at age 46–50; after this age range, the incidence is about 20% from age 51 to 65.

The highest rate of cancer among less-developed countries such as Bangladesh is in the male population 46 to 70 years old, and over the age of 70, it decreases, based on a survey conducted from 2012 to 2015. Benign prostate cancer is more common than malignant prostate cancer. The rate of prostate cancer-related deaths in Bangladesh increased to 1.5%, and the total number of deaths was 773 in 2018 [59]. For prostate cancer detected early, surgery is the most common treatment. Most patients were hospitalized due to urinary tract infection. Common symptoms included blood in the urine, enlarged prostate, frequent urination, and changes in the color of urine. A study conducted in 2012, 2013, and 2015 indicated there were more than 130 patients in different hospitals in Bangladesh, with frequency based on age, as shown in Figure 6 [11,58].

## 4. Discussion

In this systematic review, we focused on the causes and rates of cancer in less-developed countries such as Bangladesh. The information provided here would be beneficial for governments to make policies and for people to be more conscious of this deadly disease. In summary, it can be concluded that tobacco use is the most common cause of cancer for both males and females [10]. Regular consumption of certain foods is the second most common contributing factor in oral, stomach, esophagus, liver, and breast cancer. The favorite foods among the Bangladeshi population are rice, fish, red meat, and dried fish (known as sutki). The most important parameter leading to cancer is food adulteration, particularly of dried fish (sutki) [60]. At the same time, the poor health care system, illiteracy, poverty, lack of awareness, and lack of proper diagnostic procedures are also responsible for the high rate of cancer. The detection of cancer is very difficult. In women, the most predominant type is cervical cancer, followed by breast cancer, with number of sexual partners, age, lack of sexual hygiene, and virus infection being the most common causes in less-developed countries, especially Bangladesh [60,61].

### Limitations of This Study

It was difficult to get the desired amount of data from the reputable published papers. In Bangladesh, research papers are published very slowly in unaffiliated journals, but nowadays this trend has changed. We selected the most recently published papers for statistical data analysis. Another major limitation was the need to identify predatory journals and cancel those papers from time-to-time in order to obtain the best results.

## 5. Conclusions

This study shows that different types of cancers are prevalent among less-developed countries, especially Bangladesh. Every year, the number of cancer patients among the Bangladeshi population has increased. This study also shows the causes of cancer in different age groups. The rate of lung cancer in males is high, as the smoking rate is comparatively high among young men compared to women. The incidence of cervical cancer is highest among women who have little knowledge about cancer and unhygienic sex. Breast cancer is the second most prevalent cancer in Bangladeshi women. The rates of death are higher for these types of cancer due to the late diagnosis. Early detection and awareness could reduce the mortality rate for both men and women. Establishing a database to keep the histories of patients and their family members is essential to reduce the risk and lower the death rate by early detection. Vaccination among young females can reduce the risk of uterine cancer as well.

## Figures and Tables

**Table 1:** Major causes of lung cancer among Bangladeshi population including percentage ratio based on cause and indicating chance of lung cancer in female population.

Risk Factors	Male				Female			
	Yes	Percentage	No	Percentage	Yes	Percentage	No	Percentage
Daily alcohol intake	4	4.1%	94	95.9%	0	0	6	100%
Genetic inheritance	16	16.32%	82	83.7%	0	0	6	100%
Previous history of lung disease (Asthma, TB)	31	31.6%	67	68.4%	3	50%	3	50%
Highly cooked food	41	41.8%	57	58.2%	1	16.7%	5	83.3%
Tobacco leaf intake	47	48.0%	51	52.0%	4	66.7%	2	33.33%
Smoking	87	88.78%	11	11.22%	0	0	6	100%

**Table 2:** Independent variables for breast cancer in Bangladeshi females.

General Risk Factors	Reproductive and Hormonal Factors	Anthropometric Indicators
Age and Sex	Age at menarche	Family history
Socioeconomic condition	Age at menopause	Height
Residence	Parity	Weight
Food habit	Breastfeeding	Ionizing radiation
Alcohol consumption	At the age of childbirth	Benign breast disease

**Table 3:** Breast cancer scenario, age 15–94.

Year	2005–2010	2016
Number of patients	5255	400
Age ranges	15–94	15–39
Mean age	41.8	27
Most common	15–44	35–39
Invasive ductal carcinoma	95%	82%

## Data Availability

Data will be available upon reasonable request.

## Supplementary Materials

The following are available online at https://www.mdpi.com/article/10.3390/healthcare10030424/s1, Table S1: PRISMA check list for different cancer diseases. Table S2: Comprehensive way to search terms and selection of data

## Abbreviations

DDT, Dichlorodiphenyltrichloroethane; NAFLD, Nonalcoholic fatty liver diseases; HCC, Hepatocellular carcinoma; NIRCH, National Institute of Cancer Research and Hospital; MALT. Mucosa-associated lymphoid tissue; TB, Tuberculosis; HIV, Human immunodeficiency virus.
