# Supplementary material for: Comprehensive Studies of Different Cancer Diseases among Less-Developed Countries
Source: Healthcare (Basel). 2022 Feb 24;10(3):424. doi: 10.3390/healthcare10030424 (PMC8949682; doi:10.3390/healthcare10030424)
Supplement: Supplementary file 1 [file healthcare-10-00424-s001.zip › healthcare-1542651-supplementary.pdf]

Table S1: PRISMA check list for different cancer diseases

	Item #	Checklist item	Location where item is reported
**TITLE**			
Title	1	Identify the report as a systematic review.	Page 1, line 1
**ABSTRACT**			
Abstract	2	See the PRISMA 2020 for Abstracts checklist.	Page 1
**INTRODUCTION**			
Rationale	3	Describe the rationale for the review in the context of existing knowledge.<br>Response: Please find this on the research paper. “However, in Bangladesh lung cancer is more common in men, cervical and breast cancer in females almost 38 percent of all cancer (T, None and J, 2008). Most cases of cervical carcinoma are being diagnosed at the late stage in Bangladeshi females (Uddin et al., 2013). So, the rate of cancer survival rate is low due to late diagnosis (A et al., 2011) such as cervical cancer (T, None and J, 2008; Ahmed et al, 2008). In 2010, 55% of people were affected and 9 million may die by 2030 in this disease (Uddin et al., 2013)”	Page 1-2
Objectives	4	Provide an explicit statement of the objective(s) or question(s) the review addresses.<br>Response: In our study, it has been focused that the cancer rate among Bangladeshi people with the cause behind this.	Page 2
**METHODS**			
Eligibility criteria	5	Specify the inclusion and exclusion criteria for the review and how studies were grouped for the syntheses.<br>Response: We try to find the all the data from the journal included in google scholar and in PubMed. Study groups were selected based on the different data collected from hospital	Page 3
Information sources	6	Specify all databases, registers, websites, organisations, reference lists and other sources searched or consulted to identify studies. Specify the date when each source was last searched or consulted.<br>Response: All the data bases, websites, and sources of data collection were mentioned in reference section. From any website or news paper data collection we mentioned in the reference part.	Page number 18,19
Search strategy	7	Present the full search strategies for all databases, registers, and websites, including any filters and limits used.<br>Response: Search in google by content like colon, breast cancer in lower income countries like Bangladesh. Filter the Indian predatory journal and find the journal find it on the google scholar.	Supplementary Table 2
Selection process	8	Specify the methods used to decide whether a study met the inclusion criteria of the review, including how many reviewers screened each record and each report retrieved, whether they worked independently, and if applicable, details of automation tools used in the process.<br>Response: Each paper has been cited for different journals several reviewers reviewed before publication as this paper are highly cited. For our paper writing three person we screened data separately	Page number 2
Data collection process	9	Specify the methods used to collect data from reports, including how many reviewers collected data from each report, whether they worked independently, any processes for obtaining or confirming data from study investigators, and if	Page 2

	Item #	Checklist item	Location where item is reported
		applicable, details of automation tools used in the process.<br>Response: I found the journal where several numbers of patients has been selected based on age sex and from several hospitals from Bangladesh the author get the data. We three person collected data independently and then accumulated	
Data items	10a	List and define all outcomes for which data were sought. Specify whether all results that were compatible with each outcome domain in each study were sought (e.g., for all measures, time points, analyses), and if not, the methods used to decide which results to collect.<br>Response: All results were shown in graph and in table. All data has been taken by searching specific cancer and the results has been provided in good journal	Find the table and graph
	10b	List and define all other variables for which data were sought (e.g., participant and intervention characteristics, funding sources). Describe any assumptions made about any missing or unclear information.<br>Response: The authentication has been provided by talking with the author of journal also all the paper are renowned for their publication. No assumption has been made.	
Study risk of bias assessment	11	Specify the methods used to assess risk of bias in the included studies, including details of the tool(s) used, how many reviewers assessed each study and whether they worked independently, and if applicable, details of automation tools used in the process.<br>Response: The chance of misleading results for my review is zero as I selected all data from highly renowned and cited journal	All cancer cause and prevalence
Effect measures	12	Specify for each outcome the effect measure(s) (e.g., risk ratio, mean difference) used in the synthesis or presentation of results.<br>Response: The average value has been taken for few cases to show the results in table or graph	Table1, Table 4
Synthesis methods	13a	Describe the processes used to decide which studies were eligible for each synthesis (e.g., tabulating the study intervention characteristics and comparing against the planned groups for each synthesis (item #5)).<br>Response: Each case the number of affected people were determined based on age, sex, or their daily habit	Page 09,11,14
	13b	Describe any methods required to prepare the data for presentation or synthesis, such as handling of missing summary statistics, or data conversions.<br>Response: We modified data and rigorously scanned again and again to find the proper results, so no data conversion is not necessary	
	13c	Describe any methods used to tabulate or visually display results of individual studies and syntheses.<br>Response: Microsoft excel was used to make graph	Table 1

PRISMA 2020 Checklist

	Item #	Checklist item	Location where item is reported
	13d	Describe any methods used to synthesize results and provide a rationale for the choice(s). If meta-analysis was performed, describe the model(s), method(s) to identify the presence and extent of statistical heterogeneity, and software package(s) used.<br>Response: The results were collected from several published journals by keeping in Microsoft excel, anova software analysis	Page 2
	13e	Describe any methods used to explore possible causes of heterogeneity among study results (e.g., subgroup analysis, meta-regression).<br>Response: As far my knowledge the data variability did not observe in my paper.	
	13f	Describe any sensitivity analyses conducted to assess robustness of the synthesized results.<br>Response: For result analysis no data has been taken that me arise confliction	
Reporting bias assessment	14	Describe any methods used to assess risk of bias due to missing results in a synthesis (arising from reporting biases).<br>Response: No missing result in this paper. All data has been selected from published paper	
Certainty assessment	15	Describe any methods used to assess certainty (or confidence) in the body of evidence for an outcome.<br>Response: The best way to evaluate the data search by the reference conducted in each section	Reference Section
**RESULTS**			
Study selection	16a	Describe the results of the search and selection process, from the number of records identified in the search to the number of studies included in the review, ideally using a flow diagram.<br>Response: six most common cancer has been selected in our study, especially dominated over Bangladeshi population	Page 2 to 11
	16b	Cite studies that might appear to meet the inclusion criteria, but which were excluded, and explain why they were excluded.<br>Response: No study is not provided without references	
Study characteristics	17	Cite each included study and present its characteristics.<br>Response: Most of the study included in my paper has already been cited by different papers and approved by several journal committee.	
Risk of bias in studies	18	Present assessments of risk of bias for each included study.<br>Response: No misleading results. All data has been taken from published journals.	
Results of individual studies	19	For all outcomes, present, for each study: (a) summary statistics for each group (where appropriate) and (b) an effect estimates and its precision (e.g., confidence/credible interval), ideally using structured tables or plots.	See all Tables and Figures
Results of	20a	For each synthesis, briefly summarise the characteristics and risk of bias among contributing studies.	

PRISMA 2020 Checklist

	Item #	Checklist item	Location where item is reported
syntheses		Response: No misleading results as all data has been taken form journal	
	20b	Present results of all statistical syntheses conducted. If meta-analysis was done, present for each the summary estimate and its precision (e.g., confidence/credible interval) and measures of statistical heterogeneity. If comparing groups, describe the direction of the effect.<br>Response: Statistical significance, STD were not provided just mean value has calculated.	Figures 5,4,3
	20c	Present results of all investigations of possible causes of heterogeneity among study results.<br>Response: As far my knowledge the data variability did not observe in our paper.	
	20d	Present results of all sensitivity analyses conducted to assess the robustness of the synthesized results.<br>Response: For result analysis no data has been taken that me arise confliction	
Reporting biases	21	Present assessments of risk of bias due to missing results (arising from reporting biases) for each synthesis assessed.<br>Response: No misleading results. All data has been taken from published journals.	
Certainty of evidence	22	Present assessments of certainty (or confidence) in the body of evidence for each outcome assessed.<br>Response: Each result is authentic as are published in paper	
**DISCUSSION**			
Discussion	23a	Provide a general interpretation of the results in the context of other evidence.<br>Response: The discussion included in result section.	Page 10
	23b	Discuss any limitations of the evidence included in the review.<br>Response: The limitation was included in the paper	Page 10
	23c	Discuss any limitations of the review processes used.<br>Response: The limitations is to find the more results published in highly cited journals. So most cited journal has been taken in consideration in our study	Page10
	23d	Discuss implications of the results for practice, policy, and future research.<br>Response: The data will show the risk factor of cancer, help to people more conscious and in future they will provide scientist to research more vigorously.	Page 10,11
**OTHER INFORMATION**			
Registration and protocol	24a	Provide registration information for the review, including register name and registration number, or state that the review was not registered.	
	24b	Indicate where the review protocol can be accessed, or state that a protocol was not prepared.	

PRISMA 2020 Checklist

	Item #	Checklist item	Location where item is reported
		Response: Need to find the data from published papers, or providing through email	
	24c	Describe and explain any amendments to information provided at registration or in the protocol.<br>Response: No information provided that needs to be corrected in future	
Support	25	Describe sources of financial or non-financial support for the review, and the role of the funders or sponsors in the review.	Page 11
Competing interests	26	Declare any competing interests of review authors.	Page 11
Availability of data, code and other materials	27	Report which of the following are publicly available and where they can be found template data collection forms; data extracted from included studies; data used for all analyses; analytic code; any other materials used in the review.<br>Response: Searched data in google scholar and PubMed based on reference provided	

Here, PRISMA check list for different cancer diseases are included in this section (Table S1). In this checklist, Title, Abstract, Introduction, Methods, Results, Discussion, and other information related studies are included. Each term has been elaborately mentioned in every particular section on different questionaries on the term as "Rationale, Objectives, Eligibility, Information sources, Search strategy, Selection process, Data collection process, Data items, Study risk of bias assessment, Effect measures, Synthesis methods, reporting bias assessment, Certainty assessment, Study selection, Study characterization, Risk of bias in studies, Results of individual studies, Results of synthesis, Reporting biases, certainty of evidence, Discussion, Registration and protocol, Support, Competing interests, Availability of data, code, and other materials etc".

Table S2: Comprehensive way to search terms and selection of data

Search term	Measurement	Assessment	Benefits
Diseases (Several types of cancer in Bangladesh)	Patients number, study hospital, age, sex, food habit, any disease condition, cause, number of statistical data from several hospital	All results were monitored by the authors	"Got the number of statistical data"<br>"Patient treatment condition"<br>"Recent used of drugs"<br>"Location of most affected"<br>"Important reasons behind cancer"

PRISMA 2020 Checklist

Here, authors' have searched the different types of cancer in Bangladesh in various published sources in this section, which are included in this Table (Table S2). For this purpose, patient's number, study hospital, age, sex, food habit, any disease condition, cause, number of statistical data from several hospital are considered in this review report. There are several benefits of this review evaluation, such as "Got the number of statistical data", "Patient treatment condition", "Recent used of drugs", "Location of most affected", and "Important reasons behind cancer".
